# Development of a knowledge graph framework to ease and empower translational approaches in plant research: a use-case on grain legumes

**DOI:** 10.3389/frai.2023.1191122

**Published:** 2023-08-03

**Authors:** Baptiste Imbert, Jonathan Kreplak, Raphaël-Gauthier Flores, Grégoire Aubert, Judith Burstin, Nadim Tayeh

**Affiliations:** ^1^Agroécologie, INRAE, Institut Agro, Univ. Bourgogne, Univ. Bourgogne Franche-Comté, Dijon, France; ^2^Université Paris-Saclay, INRAE, URGI, Versailles, France; ^3^Université Paris-Saclay, INRAE, BioinfOmics, Plant Bioinformatics Facility, Versailles, France

**Keywords:** graph database, orthology, ontology, quantitative genetics, gene expression, comparative omics, Ortho_KB, OrthoLegKB

## Abstract

While the continuing decline in genotyping and sequencing costs has largely benefited plant research, some key species for meeting the challenges of agriculture remain mostly understudied. As a result, heterogeneous datasets for different traits are available for a significant number of these species. As gene structures and functions are to some extent conserved through evolution, comparative genomics can be used to transfer available knowledge from one species to another. However, such a translational research approach is complex due to the multiplicity of data sources and the non-harmonized description of the data. Here, we provide two pipelines, referred to as structural and functional pipelines, to create a framework for a NoSQL graph-database (Neo4j) to integrate and query heterogeneous data from multiple species. We call this framework Orthology-driven knowledge base framework for translational research (Ortho_KB). The structural pipeline builds bridges across species based on orthology. The functional pipeline integrates biological information, including QTL, and RNA-sequencing datasets, and uses the backbone from the structural pipeline to connect orthologs in the database. Queries can be written using the Neo4j Cypher language and can, for instance, lead to identify genes controlling a common trait across species. To explore the possibilities offered by such a framework, we populated Ortho_KB to obtain OrthoLegKB, an instance dedicated to legumes. The proposed model was evaluated by studying the conservation of a flowering-promoting gene. Through a series of queries, we have demonstrated that our knowledge graph base provides an intuitive and powerful platform to support research and development programmes.

## Introduction

To accelerate plant research and manage costs, model species first emerged as a good strategy for studying plant development and stress response, thus providing the research community with data and knowledge. Databases such as TAIR for *Arabidopsis thaliana* (Berardini et al., 2015), MTGD for *Medicago truncatula Gaertn*. (Krishnakumar et al., 2015), miyakogusa-jp for *Lotus japonicus* (Sato et al., 2008) or RAP-DB for *Oryza sativa* (Ohyanagi et al., 2006) were created to centralize, organize and curate the information on model species while providing tools for their analysis. Meanwhile, researchers working on other species have been inferring information from closely-related model plants using orthology and synteny. In fact, orthologs, i.e. genes descending from a common ancestral gene by a speciation event, are likely to have similar and conserved functions (Linard et al., 2021). However, it can prove difficult to identify the correct ortholog of a gene among its homologs based only on sequence similarity because of duplication events. Synteny and collinearity, i.e., conservation of the content and the order of genes on chromosomal regions, respectively, can help identifying orthologous blocks and hence deciphering true orthologous genes (Drillon et al., 2020). Such information is already made available and exploited through platforms such as PLAZA (Van Bel et al., 2012, 2022), sometimes supplemented by tools giving access to gene expression data (Kamei et al., 2016).

With the advent of new technologies, the once daunting sequencing costs have been dramatically reduced (Shendure et al., 2017), allowing for the production of high-quality assembled genomes including for orphan species (Ye and Fan, 2021). These new resources, along with associated annotations, are often being hosted on dedicated websites and/or made available in repositories of well-known databases such as NCBI (Sayers et al., 2022), Ensembl Plants (Yates et al., 2022), Gramene (Tello-Ruiz et al., 2021) or Phytozome (Goodstein et al., 2012). The release of the genome sequences is significantly boosting the production of genetic data to inform on the control of phenotypic traits by genes and the production of -omic data (mostly represented by genomics, transcriptomics, proteomics and metabolomics) to characterize and quantify the different molecules from a biological entity. However, efforts are still uneven across the broad spectrum of species since conducting experiments spanning a wide range of genotypes, tissues and conditions to generate solid data can be very informative, but also expensive and hard to achieve. Also, some quantitative trait loci (QTL) controlling quantitative traits still display low resolution, either due to low marker density or to low recombination rate in the respective genomic regions, which can result in large number of genes within the confidence intervals and long lists of candidate genes. Comparing QTL positions across species can help pinpointing orthologous ones and thus refining the intervals of those with low resolution. Such comparative translational research has also the potential to transfer functional information from one species to another or to a group of species.

Databases are powerful tools to leverage already produced datasets, not only as a mean of storage but also of intelligent exploitation. For example, the Comparative Genomics (CoGe) platform currently allows for the comparison of datasets from a wide range of organisms, with nearly 58,000 genomes available (Lyons and Freeling, 2008). Using sequence homology and synteny, researchers can identify structural and nucleotide variations for their species of interest. Researchers can also use the LoadExp+ extension to import experimental data in various common formats, such as VCF for polymorphism or FASTQ for RNA-seq, process them, and display the results as tracks in the genome browsers (Grover et al., 2017). Nevertheless, the data are predominantly stored using relational database management system (RDBMS), distributed in category-specific tables. One problem than can arise with RDBMS is that connecting tables containing large datasets during querying requires several joining operations, which are expensive in terms of time and computational resources (Vicknair et al., 2010).

The intuitive idea of structuring intertwined data into a graph was propelled by the World Wide Consortium for semantic web through the Resource Description Framework (RDF), (W3C, 1994). In order to obtain a logical model in RDF, each piece of data is sliced into atomic statements stored as triples, i.e., (1) the subject of the resource to describe, (2) a property assigned to the resource, termed predicate and (3) the object, either a description or another resource. The subject and the object are nodes in the graph, while the predicate is an edge connecting the two nodes (Abuoda et al., 2022). The directional decomposition of information allows the use of ontologies that organize knowledge and greatly improve data sharing in scientific communities (Stevens et al., 2020). However, databases using this format, called triplestores or RDF stores, are characterized by an atomic granularity of nodes which can make database modeling tedious. In addition, deep traversal of the graph requires self-joining of all traversed triples which can make the cost of traversing edges logarithmic (Donkers et al., 2020).

Alongside RDF, labeled-property graph (LPG) databases have emerged, currently led by Neo4j, which are fundamentally designed to improve graph traversal by directly storing on disk all existing edges between nodes. A benchmark from Khayatbashi et al. (2022) comparing RDF triple-stores and LPG databases with twelve queries shows that Neo4j is in fact more efficient to traverse multiple layers of data. Neo4j databases offer high flexibility by adding key-value properties to nodes and edges to effectively compact information, consequently making the modeling easier to read and to incrementally improve (Donkers et al., 2020; Neo4j, 2023b). Considering these assets, Neo4j databases were found advantageous to manage dense networks of information required for systems biology. The Reactome database (Fabregat et al., 2018) and its plant counterpart Plant Reactome (Naithani et al., 2019) have already switched from an RDBMS database to a Neo4j database, since metabolic pathways are intrinsically connected as a graph structure. In fact, using a graph database dropped the average query time of Reactome by 93 % (Fabregat et al., 2018). While a graph is intuitive when representing a biological pathway, the value of such modeling extends to many applications, including translational research. For instance, orthologous relationships across genes required for translational research, could be modeled in a Neo4j database with an “IS_ORTHOLOGOUS_TO” relationship between the two “Gene” nodes. Information regarding the gene identifier or annotation could be stored as internal node properties, available for querying. As the system is adaptable, new layers of data can successively be added and articulated. Omics Database Generator (ODG) is the first LPG designed for translational research as defined by Guhlin et al. (2017). ODG is a Neo4j graph-database, developed primarily for annotation transfer to non-model species of bacteria and plants. The structure of ODG has been made available for researchers to import their own data. Indeed, the comparison of newly generated data with existing data can confirm hypotheses or help to generate new ones. This is especially useful when datasets do not yield results supporting the initial research hypothesis, end up being set aside and remain unpublished (Raciti et al., 2018). It is therefore crucial to use as many available and high-quality datasets as possible, whether published or unpublished, as valuable sources of knowledge. However, ODG does not offer support for the integration of annotated genetic data, which is necessary for crop improvement, and it is likely to be difficult for non-expert users to understand its model and its underlying potential (Misra et al., 2019; Kaur et al., 2021).

The legume family (Leguminosae or Fabaceae) is the third largest family of flowering plants, with about 750 genera and nearly 19,500 species (The Legume Phylogeny Working Group et al., 2013). The Leguminosae include many taxa of agricultural or other economic importance and significant research efforts are needed to advance legume breeding and address the new challenges imposed to agriculture, namely production under climate change, with less pesticides and fertilizers. *Pisum sativum* L. (pea), *Lens culinaris* Medik. (lentil) and *Vicia faba* L. (faba bean) are examples of grain legumes that produce protein-rich seeds and play a key role in sustainable cropping systems (Guiguitant et al., 2020; Rubiales et al., 2021; Semba et al., 2021). Because of their large genomes, sometimes up to 30 times larger than the genome of the model legume *M. truncatula* (Jayakodi et al., 2023), the creation of -omics data on these species has lagged behind. In addition, data on a given species were mostly produced by the research community in the country of production, as the dominant production areas are sometimes different. Several databases have been developed that attempt to inventory the diversity of published datasets and provide tools to analyse and visualize them, including Soybase (Grant et al., 2010), the Pulse Crop Database (Humann et al., 2019), KnowPulse (Sanderson et al., 2019) and the Legume Information System (Berendzen et al., 2021). However, there is still a lack of options to link multi-species datasets together for further study.

LegumeIP is a relational database, initially created to transfer knowledge from model to crop legume species, and recently transformed into an integrative platform to support translational research, hosting homology, gene annotation and expression data for 17 legume species in its latest version (Li et al., 2012, 2016; Dai et al., 2021). Some recently sequenced cool-season legumes are however missing, including *P. sativum* (Kreplak et al., 2019), *L. culinaris* (Ramsay et al., 2021) and *V. faba* (Jayakodi et al., 2023). In addition, the interface of LegumeIP is designed to facilitate pairwise comparisons, from model species to less studied crop species, making the current design unsuitable for simultaneous comparison of multi-species experiments.

Here, we developed Ortho_KB, a robust framework for translational research in diploid plant species. We developed a first pipeline to compute homology and define syntenic chromosomal regions across species. This method was chosen to identify putative orthologs among homologs, thus establishing links between corresponding genes and connecting chromosomes. We designed a second pipeline to execute custom scripts that reformat all heterogeneous data files, including -omics datasets, for input into the database. Users can integrate published and unpublished information related to their species of interest including gene-phenotype associations from QTL data and expression information from transcriptomic resources and use the provided framework to get the most out of their data. Ortho_KB provides an intuitive database model that can be queried using Cypher language, to extract meaningful information in comma-separated values (CSV) files for further analysis. The framework has been applied to a subset of legume species, resulting in a database called OrthoLegKB, a multi-species and multi-omics graph-based database for collecting, integrating and querying heterogeneous data. OrthoLegKB currently allows the comparison of genetic, and -omic data from 5 legume species, i.e., *P. sativum, V. faba, L. culinaris, Vigna radiata (L.) R.Wilczek* and *M. truncatula*. Finally, a use-case is described to demonstrate how the combination of quantitative genetics and expression data is possible in OrthoLegKB and can benefit translational research.

## Materials and methods

### Orthology and synteny

**Figure 1:**
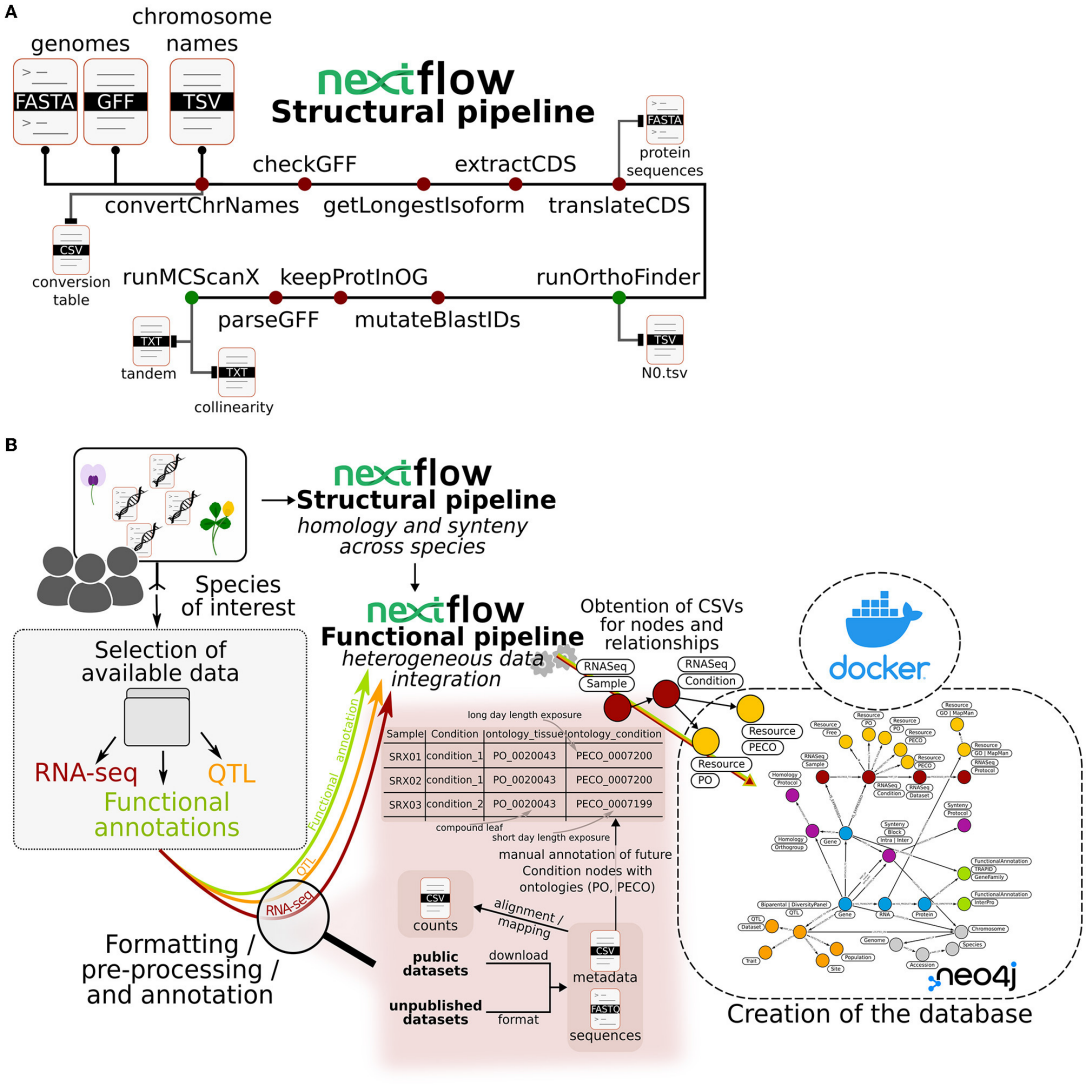
Schematic representation of the pipelines used to build Ortho_KB, a NoSQL graph database framework for translational research. **(A)** The structural pipeline computing homology between genes and synteny across chromosomal regions from selected annotated genomes. All processes included in the pipeline, except those producing the mandatory final outputs, are represented by dark red circles. Processes producing the mandatory final outputs are represented by green circles. **(B)** General overview of the steps leading to the construction of an instance of Ortho_KB. Datasets that can be managed include RNA-seq data, QTL and functional annotations. As an example, we develop the treatment of an RNA-seq dataset from public or private origin. Alongside a regular extraction of counts, metadata of the samples must be annotated using ontologies to describe in particular the tissue of origin (Plant Ontology) and the experimental conditions to which the sample was subjected to (Plant Experimental Conditions Ontology). The functional pipeline will process inputed files and in this case the annotated metadata file will produce “Sample” and “Condition” nodes in the graph. This last node will also be connected by relationships to “Resource” nodes corresponding to the ontologies, thereby conserving the metadata information in the Neo4j graph database. The graph database is included in a Docker container, as shown on the right-hand side of the schema.

As illustrated in Figure 1A, in order to identify homologous genes and syntenic regions, genome FASTA and annotation files as well as an optional conversion table for chromosomes are used as input files. The conversion table must include the original chromosome ID and the desired ID in the database. Unique chromosome and scaffold IDs across species are more convenient for querying and are also required by synteny-visualization tools such as SynVisio (Bandi and Gutwin, 2020). The steps for synteny and orthology discovery are the following: (1) curate annotation files using the agat_convert_sp_gxf2gxf.pl parser from agat v0.9.1 by automatically removing duplicated features and/or IDs, inferring missing IDs or parent features; (2) filter annotation files to keep only the longest isoform using the agat_sp_keep_longest_isoform.pl script; (3) extract coding DNA sequences (CDS) using the agat_sp_extract_sequences.pl script (Dainat et al., 2022); (4) generate protein sequences using the Seqkit v2.3.0 translation module (Shen et al., 2016); (5) submit protein sequences in FASTA format to OrthoFinder v2.5.4 with its default parameters using Diamond v2.0.12 in ultra_sensitive mode for the alignment instead of BLAST (Emms and Kelly, 2015, 2019; Buchfink et al., 2021). Finally, to connect homologous chromosomal regions, the OrthoFinder output is used to obtain syntenic blocks. First, alignment files are filtered to retain only pairs of proteins that are part of the same orthogroup. Second, these filtered alignment files are provided to MCScanX along with a merge of annotation files from all considered species (Wang et al., 2012). A minimum number of 10 genes to form a collinear block is set by default in the pipeline. All above-mentioned steps were included in a single pipeline, called the structural pipeline, using Nextflow (Di Tommaso et al., 2017).

### Functional gene annotation

Functional annotations were conducted by manually submitting CDS sequences to annotation tools either available on online platforms or to be run locally. The TRAPID online tool returned gene families, RNA families, and Gene Ontology (GO) terms associated with submitted genes (Bucchini et al., 2021). Genes that share sequence homology are gathered in gene families. “GeneFamily” nodes hold links to the Plaza website on which information regarding family-associated GO annotations and InterPro domains are available (Van Bel et al., 2022). To attribute summarized functions to genes, we assigned MapMan bins using the online Mercator4 (https://www.plabipd.de/portal/web/guest/mercator4) which resulted in a hierarchical annotation (Lohse et al., 2014; Schwacke et al., 2019). We also used eggNOG-mapper to obtain human-readable annotation and gene symbols from protein sequences (Huerta-Cepas et al., 2019; Cantalapiedra et al., 2021). A name is assigned to each gene when available in the literature. For instance, *MtrunA17Chr3g0135361* is annotated as *ELF3* for *EARLY FLOWERING 3*. Predicted proteins of each species were further annotated by locally running InterProScan v.5.53 with the “iprlookup” option (Jones et al., 2014; Blum et al., 2021), notably using databases such as Pfam (Mistry et al., 2021), Gene3D (Lees et al., 2012) or PANTHER (Mi and Thomas, 2009).

### Genetic data extraction

The exact set of mandatory and optional information required to describe QTL data in Ortho_KB are described in the documentation available on the dedicated Git repository (see “Data availability statement” section). Briefly, the identifier, the trait name, and the associated markers are essential. A QTL arising from a study on a biparental mapping population is defined by a physical position on a chromosome between two flanking genetic markers and a peak marker within the confidence interval, if the information is available. A QTL from a genome-wide association study (GWAS) analysis is defined by a single marker location on a chromosome corresponding to the peak marker unless linkage disequilibrium data are available, then data is processed similarly to a QTL from a biparental population. Therefore, a QTL record might have information for one up to three markers. QTL data in the current version of OrthoLegKB were collected from published research articles (Supplementary Table S3).

### Transcriptomic data extraction and expression quantifications

RNA-seq datasets were manually selected from NCBI. Sample IDs associated within a BioProject were collected in each case using esearch from entrez-direct v16.2 (Kans, 2013). The sample list was fed into nf-core/fetchngs pipeline v1.7 with the option “nf_core_pipeline rnaseq” to obtain all FASTQ files along with a metadata file (Patel et al., 2022). The nf-core/rnaseq v3.8 pipeline (Patel et al., 2023) was then run with the genome files, metadata file and FASTQ files with the arguments “skip_alignment”, “pseudo_aligner salmon” and “salmon_quant_libtype A” to automatically assess strandedness (Patro et al., 2017). Salmon result files were finally processed into matrices for downstream analyses using tximport (Soneson et al., 2016). The “salmon.merged.gene_counts.tsv” file containing read counts and the “salmon.merged.gene_tpm.tsv” file containing the Transcript Per Million (TPM) normalized quantification were used for further processes. Samples listed in the metadata file originating from the nf-core/fetchngs pipeline were manually annotated to indicate the tissues used, the environmental conditions applied, and the experimental area (field, greenhouse, etc.), using the Plant Ontology (PO) and the Plant Experimental Condition Ontology (PECO) (Cooper et al., 2018).

### Database construction and implementation

#### Graph database conceptualization

The current release of Ortho_KB was built as a NoSQL database framework to store and display data in a graph structure, using the Neo4j Community Edition v4.4.18 (Neo4j, 2023b).

We chose the Neo4j graph-database management system because of (1) its efficiency in handling highly connected data, (2) the graph algorithms already implemented and (3) the expressive Cypher query language it uses and (4) its capacity to import/export data using semantic web technologies. Entities, also called nodes, and edges, also referred to as relationships, were designed in a way to carry the biological information. Each gene or transcript is represented by a node and each gene is linked to its corresponding transcript by a relationship (e.g., gene *A* has a transcript RNA *A1*). Multiple properties can be stored and queried on nodes (e.g., RNA *A1* sequence length) and relationships (e.g., position of a protein domain on the protein sequence). In addition, one or more labels can be applied to nodes to group them into a set to facilitate querying. In this paper, labels are indicated by double quotation marks, for instance the “RNA” label for nodes of transcripts.

#### Input files processing

A Nextflow pipeline called functional pipeline, was created to process heterogeneous data from the previously described sources. The pipeline requires genome files, functional annotation files, RNA-seq files and QTL files to run. For the functional annotations, the pipeline includes a set of scripts to filter and format them into nodes and relationships, following the database model. For the GO annotations obtained from TRAPID, by default, only the most specific GO terms are retained for each gene by selecting those with parameter “is_hidden” equal to 0, resulting in a 90% reduction in the number of GO terms directly associated to genes. The GO W3C Web Ontology Language (OWL) file is downloaded and parsed to import the “is_a” and “part_of” predicates as relationships in the graph to allow graph traversal (W3C, 1994). Similarly, the provided annotation files from MapMan are used to create an ontology in TURTLE syntax using rdflib v.4.2.2 (Grimnes et al., 2023). For RNA-seq, salmon pseudo-counts are by default filtered to retain only genes for which the sum of TPM across samples is >5, to avoid creating many relationships for non-expressed genes. Gene expressions in all samples from the same condition are averaged, and both arithmetic and geometric means are stored on the edge between the “Gene” and the “Condition” nodes. For genetic data, previously formatted files are processed to identify genes included in the confidence interval of QTL using pybedtools v.0.9.0 (Quinlan and Hall, 2010; Dale et al., 2011).

Briefly, for all processes, the pipeline creates CSV files to populate the database and a summary file listing all CSV files to be imported in a format readable by Neo4j. All nodes and relationships that can be generated are described in Supplementary Tables S2, S3.

#### Database implementation

A Bash script was written to create and populate the database. It includes three steps. The first step prepares the import environment by building a Docker container. Running the Docker container will start the database, by default available at http://0.0.0.0:7474/browser/. The second step performs the import to populate the Neo4j database using the neo4j-admin import command. The third step imports the PO, PECO, GO and MapMan ontologies using the n10s.onto.import.fetch method from the neosemantics (n10s) plugin (Barrasa, 2022). The import creates a node per term, connected to broader terms by a “SCO” relationship obtained from the property rdf:subClassOf. The nodes of the resulting subgraph are then labeled according to their source (PO, PECO etc.) and connected to the rest of the graph using a set of Cypher queries.

#### Plant species selection for OrthoLegKB

For this study, five species were chosen. These include the model legume *M. truncatula*, three cool-season legumes of agronomic importance, i.e., *P. sativum, L. culinaris, V. faba*, and a relatively distant warm-season legume species, *V. radiata*. All species belong to the Galegoids subclade, with the exception of *V. radiata*, which is part of the sister group, the Milletoids sub-clade. We selected the latest genomic data from *P. sativum* cultivar Cameor v.1 assembly (Kreplak et al., 2019), *M. truncatula* accession A17 v.5 assembly and v.1.9 annotation (Pecrix et al., 2018), *V. faba* accession Hedin/2 v.1.0 assembly (Jayakodi et al., 2023), *L. culinaris* cultivar CDC Redberry v.2.0 assembly (Ramsay et al., 2021) and *V. radiata subsp. radiata* cultivar VC1973A v.6 assembly (Ha et al., 2021). All genomes were assembled into chromosomes, generated using long-read technology, except for *P. sativum*. The *M. truncatula* annotation file was filtered to keep only features from EuGene and BioFileConverter. Gene prefixes were also modified using a custom script. Details on selected genome assemblies and genome statistics are available in Table 1.

**Table 1:** Specifications of species included in OrthoLegKB.

**Species**	**Genotype**	**Assembly size (Mb)**	**Number of chromosomes**	**Protein coding genes**	**Assembly references**
*Lens culinaris*	CDC Redberry	3,760	7	58,243	Ramsay et al., 2021
*Medicago truncatula*	A17	430	8	44,626	Pecrix et al., 2018
*Pisum sativum*	Cameor	3,920	7	46,905	Kreplak et al., 2019
*Vicia faba*	Hedin/2	11,900	6	34,221	Jayakodi et al., 2023
*Vigna radiata*	VC1973A	476	11	30,882	Ha et al., 2021

Values for “Protein coding genes” take into account a single isoform per gene.

### Data visualization

The visualization of the graph model was created using Arrows (Neo4j, 2023a). The UpSet plot was created using UpSetR (Conway et al., 2017). Visualization of large-scale synteny was performed with the SynVisio online tool (Bandi and Gutwin, 2020) or with tailored R scripts, while microsynteny was plotted using the R package gggenomes (Hackl and Ankenbrand, 2023).

### Hardware and query time

The server hosting OrthoLegKB is based on an OpenStack infrastructure, with 4 virtual CPUs and 8 Gb of RAM. For each query presented in the Results section, the average response time over five iterations was indicated.

## Results

### Ortho_KB is a framework for translational research in plant species

Studying a particular trait or gene often requires the collection of different types of information available on different websites and databases, for the species of interest as well as for close species. We have created Ortho_KB, a database framework built with successive pipelines to facilitate the exploration of all data relevant to a trait or gene of interest in a single environment. Ortho_KB provides a unique and multi-functional structure that can be populated with datasets of interest and then queried for comparative and functional genomics studies. The current Ortho_KB modeling aims at enabling translational research across a wide range of selected species by making data easily searchable and the process more straightforward. The framework relies heavily on orthology and synteny relationships to build bridges between species, and transfer and/or compare genetic and genomic information between them. A Nextflow pipeline, called the structural pipeline (Figure 1A), first identifies groups of homologous genes – orthogroups – based on protein sequence similarity. It then looks for conserved gene order between pairs of chromosomes, within or between species, to highlight collinear regions. Homologs in collinear regions are more likely to be orthologs and therefore have similar functions. A second Nextflow pipeline, called the functional pipeline, connects information from the first pipeline and additional data available from separate tables including gene annotation, gene expression and QTL positions (Figure 1B). All heterogeneous data are thus properly formatted for integration into the database.

### Ortho_KB uses Neo4j graph-database management system

**Figure 2:**
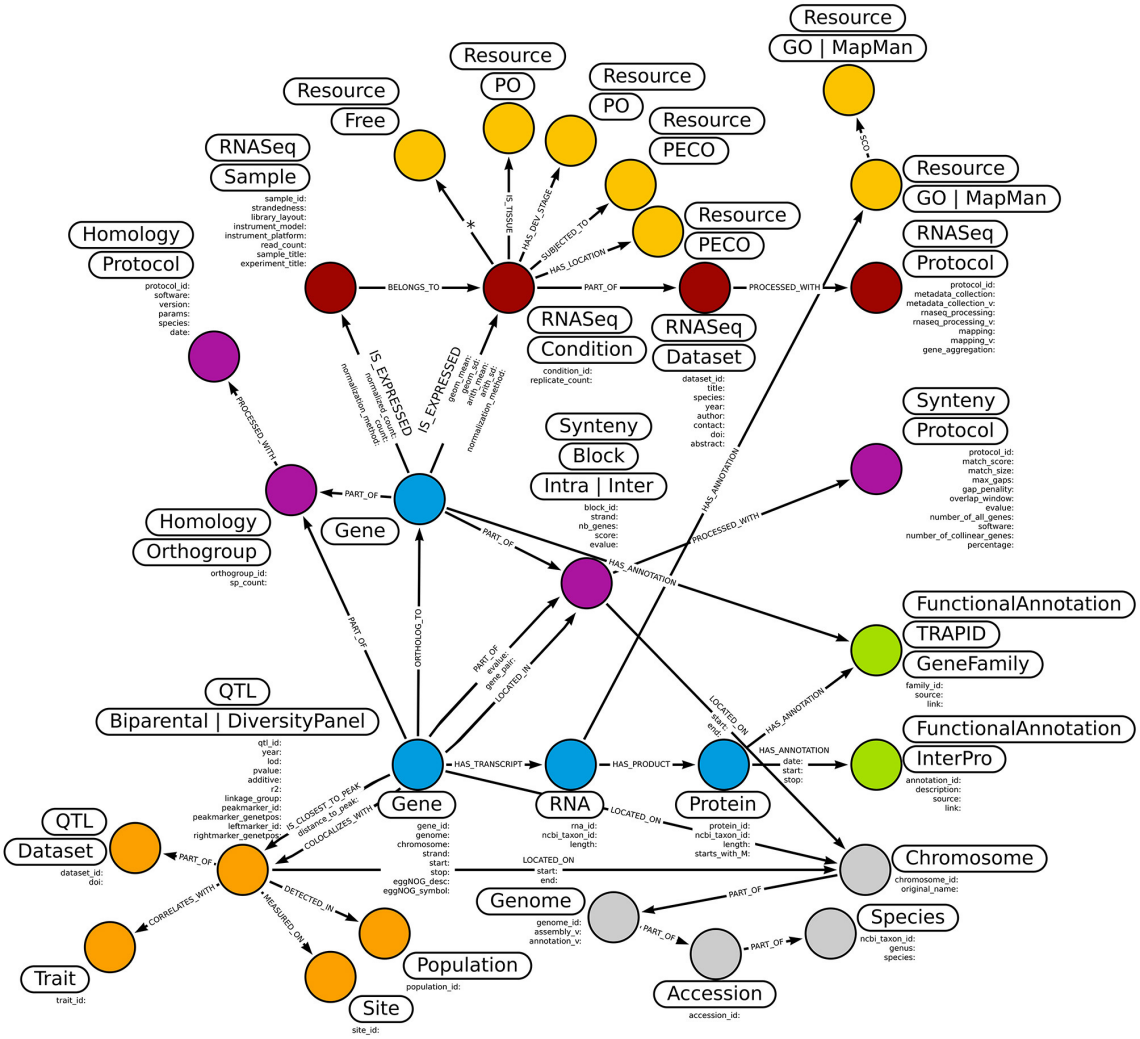
Overview of the Ortho_KB translational database model. In the graph model, colored circles represent the 29 core node types, which are entities with labels and properties. “Gene”, “RNA”, and “Protein” and related genomic nodes are shown in blue, “Homology” and “Synteny” and related nodes in mauve, ontology term nodes in yellow, the RNA-seq nodes in dark red, functional annotation nodes in light green, taxonomic nodes in light gray, and QTL-related nodes in orange. The category of each node is described by the associated labels, which are contained in elongated boxes near the nodes, and the properties correspond to the lists of elements placed below the labels. Nodes are connected to each other by relationships, represented by arrows, which can also store information as properties.

The Neo4j graph-database management system handles entities as nodes and their connections as relationships. In Ortho_KB, the data model revolves around “Gene” nodes, characterized by their start and stop positions on chromosomes (Figure 2). The “Gene” nodes are connected to their putative transcript (“RNA”) nodes, themselves connected to the predicted proteins (“Protein”) resulting from the translation of their RNA sequences. Homology and collinearity information computed using the structural pipeline create bridges across species at the gene and the chromosome levels, respectively. The current version of Ortho_KB includes 29 core categories of nodes tagged either by a single label, like “Gene” nodes or by a set of labels, like “RNASeq” supplemented by “Condition”. They are connected by directed relationships, sometimes bearing additional properties (Figure 2). Individual nodes are defined by a unique identifier. For example, a “Gene” node is defined by a gene ID, matching the feature ID from the General Feature Format 3 (GFF3) annotation file, unique across species. Ortho_KB can be queried through the web Neo4j Browser, the terminal or other interfaces provided by Neo4j (Neo4j, 2023b).

### Ortho_KB integrates different categories of data including gene annotation, genetic and transcriptomic resources

As shown in Figure 1, Ortho_KB gathers different categories of data.

In terms of functional annotation, complementary information sources are handled. These include TRAPID's gene families, GO annotations, MapMan bins and InterPro that are each integrated in a separate node type. TRAPID gene families and Mapman bins provide synthetic overviews of gene functions while GO annotations and InterPro provide detailed descriptions focusing on gene functions and protein domains, respectively (Figure 2).

Regarding genetic data, the model includes connections between genes and QTL information either resulting from QTL mapping in biparental populations or GWAS in diversity panels. Since the two mapping approaches are grouped with the “QTL” label, we added a second label, either “BiparentalPopulation” or “DiversityPanel” to differentiate them. All genes located within the confidence interval of a QTL are connected to the “QTL” node with a “COLOCALIZES_WITH” relationship. The closest gene to the peak marker is additionally connected to the “QTL” node with a “IS_CLOSEST_TO_PEAK” relationship with its distance to the peak marker included as a property. Additional information to describe a QTL are included in connected nodes such as the experimental geographical “Site”, the studied “Population” and the “Trait” (Figure 2).

For transcriptomics, we have developed scripts to handle read counts. Read counts can either be generated using the pipeline of Patel et al. (2023) to optimize comparability of data (see “Materials and methods” section), or according to the method chosen by the user. The user is also free to integrate data previously analyzed with other methods. Replicates originating from the same biological condition are summarized into a condition that has to be manually annotated with ontology terms describing best the experimental conditions and biological material. The PECO and PO ontologies were selected for this purpose (Cooper et al., 2018). Using n10s inference, this model allows to traverse the ontology and unveil datasets from experiments performed in similar conditions (Barrasa, 2022). If no ontologies are available to appropriately describe conditions, free terms might be introduced (Figure 2).

### OrthoLegKB was developed with Ortho_KB to provide a translational tool for grain legumes

To prove how Ortho_KB can serve translational approaches and the research goals of a scientific community, we chose to apply it to five diploid legume species belonging to the Galegoid (cool-season legumes) and Milletoid (warm-season legumes) clades creating the OrthoLegKB database.

**Figure 3:**
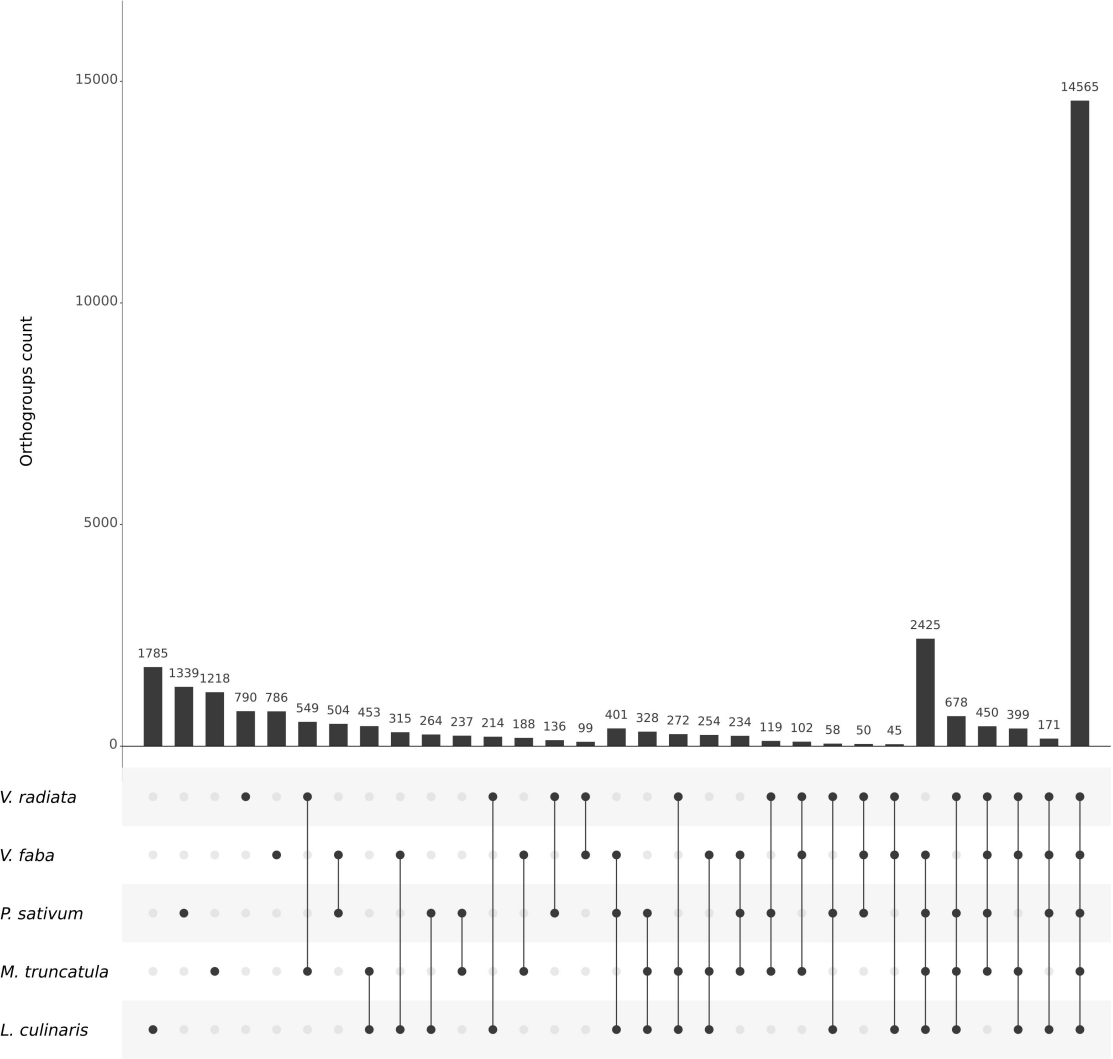
UpSet plot highlighting the number of orthogroups within and between legume species included in OrthoLegKB. The structural pipeline of Ortho_KB was used to identify the orthogroups. The bar plot shows the number of orthogroups for each possible set of species. The dots indicate the species associated with each bar.

To leverage data from all five species using comparative genomics, we started by searching for orthologs with the structural pipeline using the latest genome assemblies. The pipeline was run for 740 CPU hours with 20 CPUs allocated (7 h 40 in real time), with a maximum physical memory usage of 46 Gb. In total, 14,565 out of 29,428 total orthogroups (49.49 %) were shared by all five species and 8.24 % by all species excluding *V. radiata*, the only representative of the Milletoid clade (Figure 3).

Then, public datasets with QTL and RNA-Seq data were mined, annotated with the ontologies used in Ortho_KB and included in the database using the functional pipeline. The pipeline was run for 11 CPU hours (14 min in real time), with a maximum physical memory usage of 2 Gb. A list of these datasets is available in Supplementary Table S1. OrthoLegKB currently contains more than 815,000 nodes and close to 15,000,000 relationships associated to the different types of data. The exact number of nodes in each category can be found in Supplementary Table S4.

### OrthoLegKB can be used to address various scientific questions including the conservation of the control of flowering time in legumes

**Figure 4:**
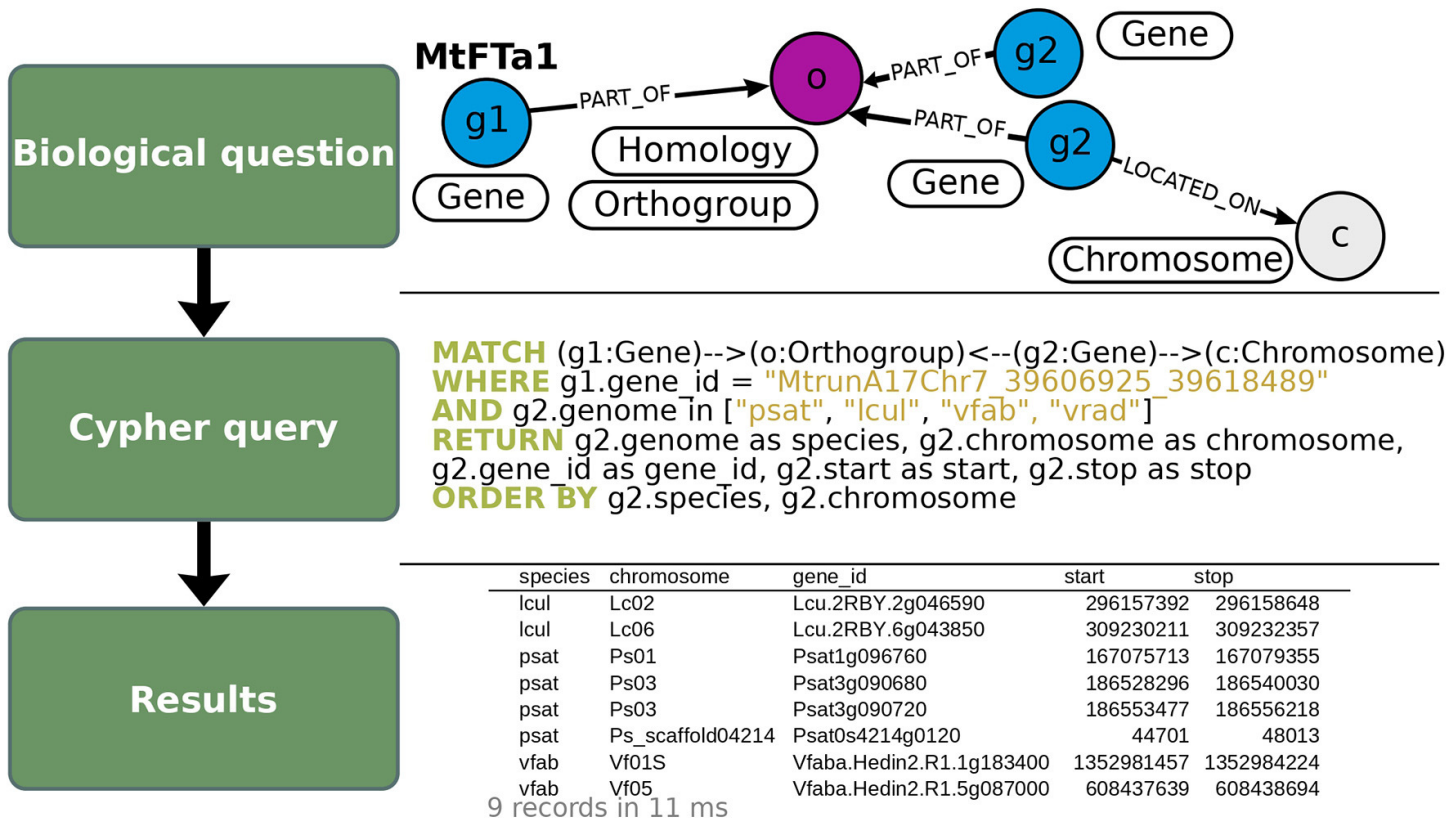
Illustration of the query used to search for putative orthologs of *MtFTa1* in OrthoLegKB. Putative orthologs in pea (psat), lentil (lcul), faba bean (vfab) and mung bean (vrad; **top panel**) were queried in Cypher **(middle panel)**, and several properties were returned in CSV format **(bottom panel)**. Genes belonging to the same orthogroup as *MtFTa1* were selected and their positions on the respective chromosomes were returned. Note that relationships' names were not displayed in the query section to keep it concise but were specified when running the query. The number of records returned in the output table and the average response time of the query are shown in light gray below the table.

**Figure 5:**
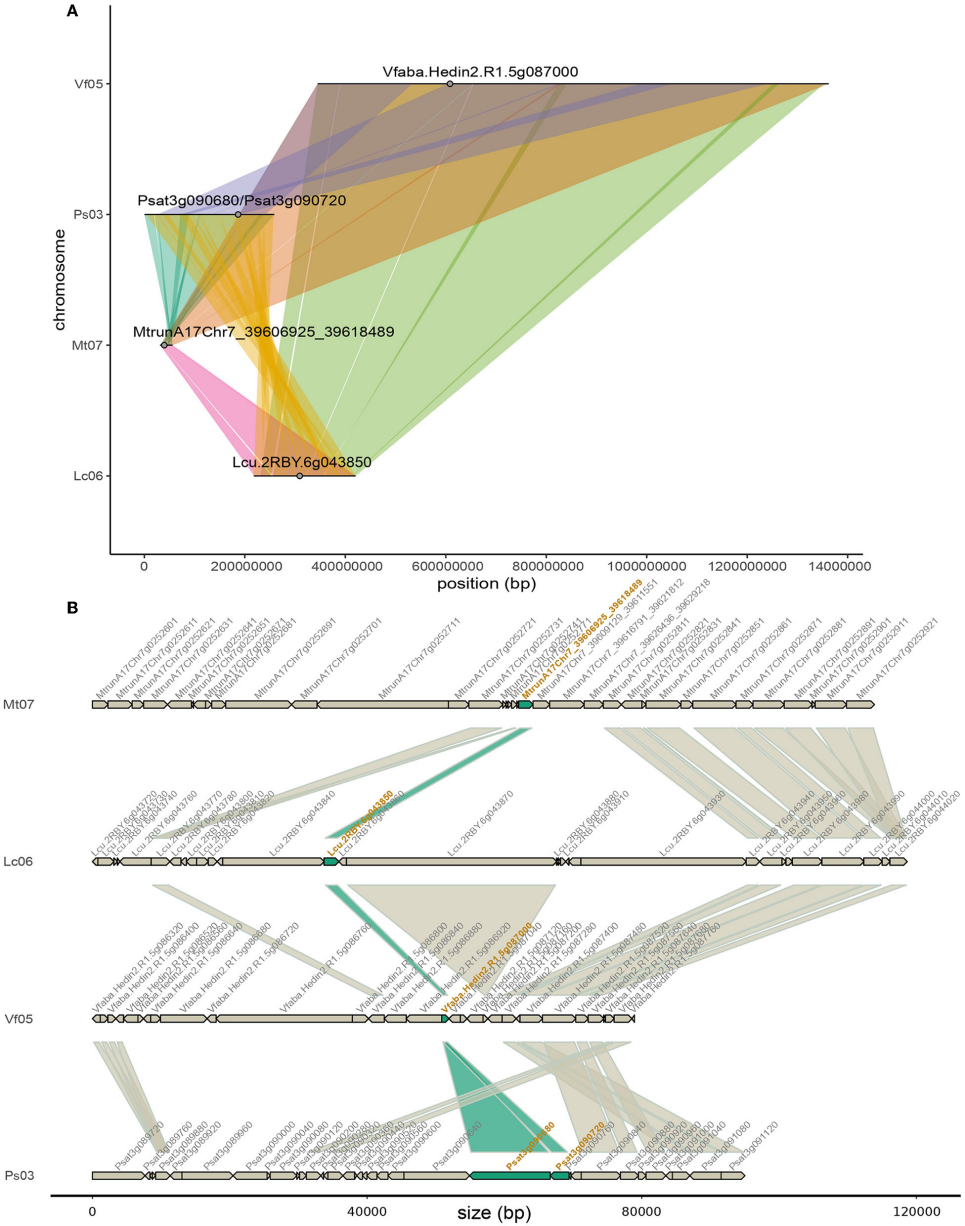
Macro- and micro-synteny of the chromosomal regions harboring *FTa1* or its orthologs in *M. truncatula, P. sativum, L. culinaris* and *V. faba*. **(A)** Macro-synteny at the chromosome level. *FTa1* and its orthologs are represented by gray dots on syntenic chromosome sections depicted as lines. Synteny between chromosomes is represented by ribbons. The positions of the two orthologs from *P. sativum* are shown even though they do not belong to any syntenic block in the database. **(B)** Micro-synteny of the *FTa1* loci. Genes are represented with arrows indicating the orientation of the open reading frames. Ribbons connect orthologous gene pairs. The IDs of *FTa1* orthologous genes are in orange and ribbons connecting them are filled in dark green. Since the four species have high genome size heterogeneity and variable intergenic sizes, intergenic regions were removed from the plot. Some gene names are not displayed due to space limitations. However, the gene sizes remain proportional.

**Figure 6:**
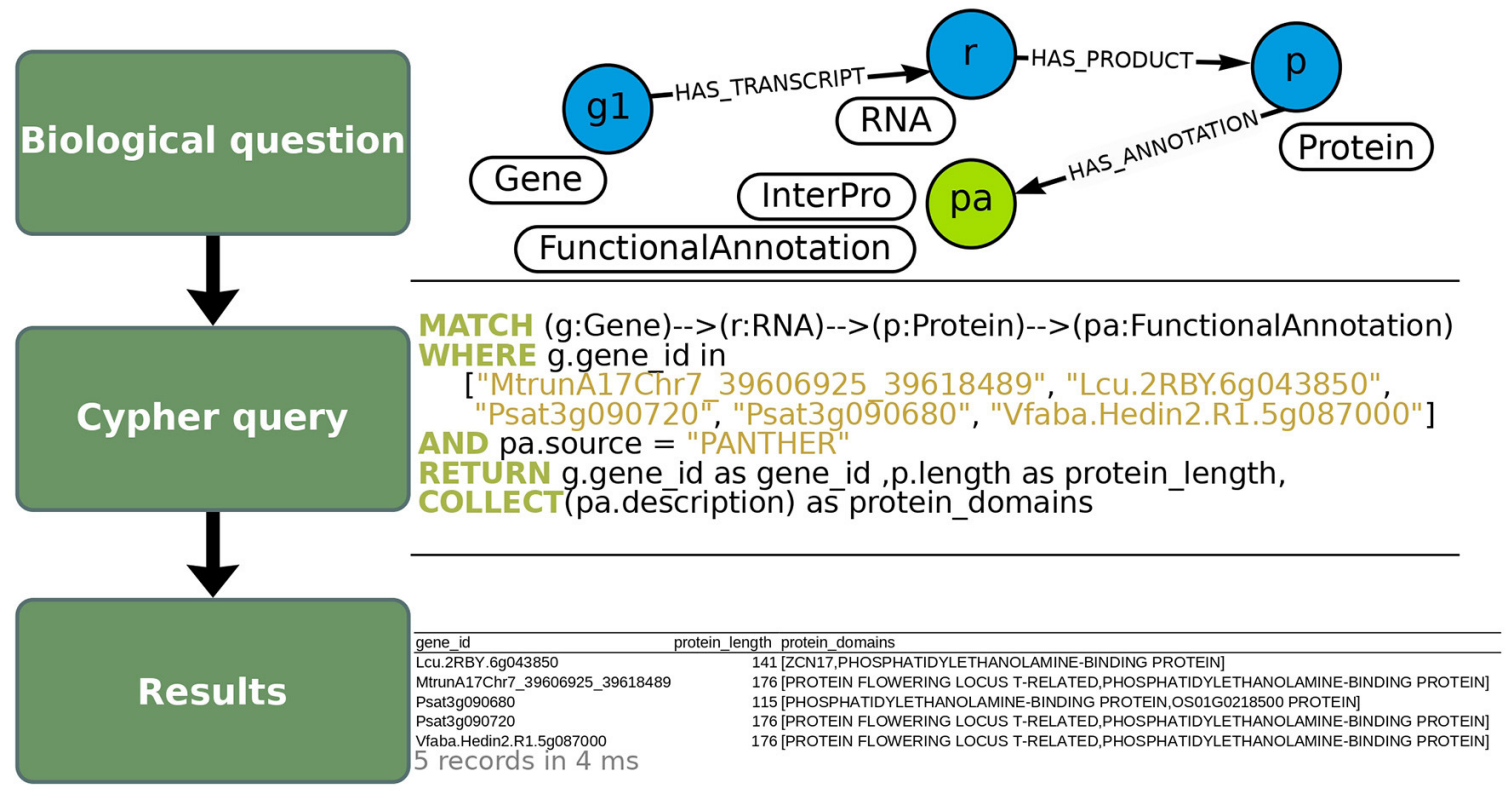
Extraction of protein domain annotations of *FTa1* and its orthologs using OrthoLegKB. “FunctionalAnnotation” nodes containing protein domain annotations **(top panel)** were queried in Cypher **(middle panel)**, for which several properties were returned in CSV format **(bottom panel)**. The nodes of protein domain annotations are connected to “Protein” nodes. Therefore, proteins corresponding to *FTa1* and its orthologous genes were selected, and their annotations from PANTHER were retrieved. Note that some relationships' names were not displayed in the query section to keep it concise but were specified when running the query. The number of records returned in the output table and the average response time of the query are shown in light gray below the table.

**Figure 7:**
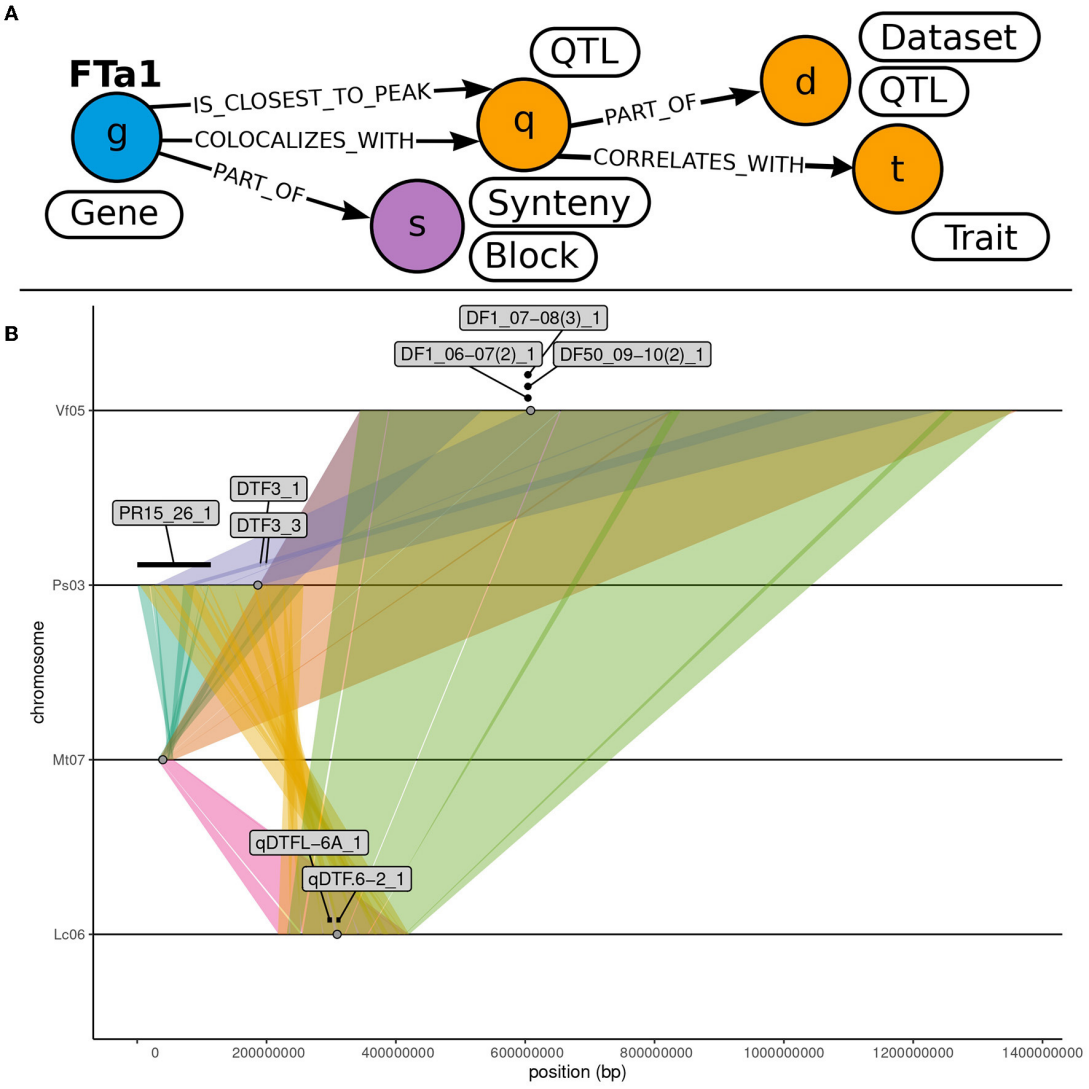
Identification of colocalising QTL with syntenic blocks hosting *MtFTa1* and its orthologs. **(A)** Illustration of the subgraph of OrthoLegKB queried to highlight QTL located near *FTa1* genes. “QTL” nodes contained within “Synteny” nodes including the *FTa1* gene were mined. Only QTL associated with flowering “Trait” were then kept. The query is available in Supplementary File S1. **(B)** Visualization of the colocalization between flowering QTL and syntenic blocks containing *FTa1* orthologs. Chromosome sections are represented by lines. Syntenic regions across chromosomes are represented by colored ribbons. *FTa1* and its orthologs are represented by gray dots. QTL labeled with their IDs are depicted by segments when information on both flanking markers is available or otherwise by simple dots.

**Figure 8:**
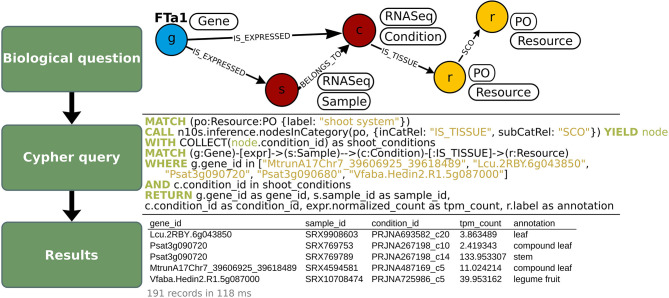
Fetch of expression levels (in TPM) of *MtFTa1* and its orthologs in different tissues of the shoot system, in OrthoLegKB. Normalized expression level of *FTa1* and its orthologs in RNA-seq samples **(top panel)** were queried in Cypher **(middle panel)**, and several properties were returned in CSV format **(bottom panel)**. The expression of *FTa1* genes was queried at the “Sample” level using the “expr” variable. The tissue annotations from the “Condition” nodes connected to these “Samples” were filtered to have only “Condition” nodes connected to the “PO” node “shoot system” or any of its more specific child terms. Note that in the table, the order of the rows was rearranged to show the diversity of annotations under the “shoot system” term. The number of records returned in the output table and the average response time of the query are shown in light gray below the table. The full table is available as Supplementary Table S6.

As a use-case to demonstrate how to exploit OrthoLegKB, we searched for the orthologs of a previously-studied flowering time regulator, the *FLOWERING LOCUS T* from *M. truncatula* (*MtFTa1*) and sought evidences for potential conserved function across species. For this use-case, we have decided to work only on cool-season legume species. *MtFTa1* has been thoroughly studied (Hecht et al., 2007, 2011; Laurie et al., 2011; Cheng et al., 2021) and its physical position on *M. truncatula* chromosome 7 (Mt07) is known. It is identified as *Medtr7g084970* (Laurie et al., 2011; Cheng et al., 2021) or *MtrunA17Chr7_39606925_39618489* in the GFF3 of the Mt5.0 (r1.9) genome annotation version. The first step was the identification of candidate orthologous genes from *P. sativum, L. culinaris* and *V. faba*. Several candidates could be found across chromosomes through a single query in 11 ms (Figure 4). OrthoLegKB was then searched for syntenic blocks encompassing these candidate genes. Synteny between chromosome 3 from *P. sativum* (Ps03), chromosome 6 from *L. culinaris* (Lc06), chromosome 5 from *V. faba* (Vf05) and Mt07 at the *MtFTa1* locus was revealed highlighting the orthologs (Figure 5A). The syntenic blocks in *L. culinaris* and *V. faba* displayed each one orthologous *FTa1* gene, while two possible orthologous genes were detected in *P. sativum* namely *Psat3g090720* and *Psat3g090680* (Figure 5B). According to the conservation of protein length and domains' annotation information from the PANTHER database stored in OrthoLegKB, *Psat3g090720* seemed to be more similar to *MtFTa1* (Figure 6). In fact, *Psat3g090680* corresponds to *FTa2* described in Hecht et al. (2011). To examine any possible links with flowering control and thus function conservation, we searched for all QTL related to flowering contained in the previously identified syntenic blocks, allowing to also return QTL that did not include *FTa1* genes in their confidence intervals (Supplementary Table S5). As depicted in Figure 7, the query identified three QTL from Aguilar-Benitez et al. (2021) on Vf05, located at the same nucleotidic positions, that were linked to the number of days from the sowing until 50 % of the plants had visible open flowers (DF50_09-10(2)_1) and the number of days from the sowing until the appearance of the first flower (DF1_07-08(3)_1 and DF1_06-07(2)_1). On Ps03, a QTL from Gali et al. (2018) corresponding to the number of days to flowering (PR15_26_1) was found upstream of the *FTa1* locus (2018). Two QTL from Williams et al. (2022), closer to the *P. sativum* locus and associated to the number of days to flowering (DTF3_1) and number of nodes on the main stem to the first flower in long days (DTF3_3) were also identified. The *L. culinaris FTa1* gene was the only gene to be part of the confidence interval of a flowering-related QTL, qDTF.6-2_1. qDTF.6-2_1 is a number of days to flowering QTL from Haile et al. (2021), and close to the qDTFL-6A_1 from Yuan et al. (2021) related to the number of days to flowering under low red/far-red light quality. Regarding expression, the *MtFTa1* gene is known to be mainly expressed in leaves and stems in *M. truncatula* (Laurie et al., 2011; Thomson et al., 2019). Therefore, we sought to investigate the top three tissues from the shoot system in which its orthologs were mostly expressed. Thanks to the inference allowed by the annotation of conditions with ontologies, we show that in the collection of RNA-seq datasets available for *M. truncatula* in OrthoLegKB, *MtrunA17Chr7_39606925_39618489* was mainly expressed in vegetative shoot apex, reproductive shoot apex and vegetative shoot system. *Psat3g090720* was expressed in the peduncle, stem and leaf tendrils. Transcripts from *Vfaba.Hedin2.R1.5g087000* were predominantly detected in adult vascular leaves, pods, and stems. For *L. culinaris*, all the experiments integrated were performed on leaves, in which the expression of *Lcu.2RBY.6g043850* was detected (Figure 8) and more particularly under far-red light conditions.

## Discussion

This paper presented the use of knowledge graphs to integrate genetic and -omics data with the aim of facilitating translational research. The main philosophy was to provide a single environment where heterogeneous datasets from multiple species can be accessed and examined with quasi-instantaneous querying time, thus allowing to address relevant biological questions, generate hypotheses, and transfer information from a single or group of species to others. The current version of the framework handles genome annotations, QTL and transcriptomic data. Users can identify orthologs, highlight candidate genes for specific traits, pinpoint possible pleiotropy and reveal conserved functional synteny. Ortho_KB gives the opportunity to capitalize on both published and unpublished datasets for further valorisation. The interest of such a database was demonstrated by populating Ortho_KB to create OrthoLegKB, a database dedicated to research on legume crop species, and supported by a use-case study focusing on a flowering-time gene.

### Ortho_KB leverages recent analytical workflows and ontology standards to host high-quality data and ensure comparability across datasets

The Ortho_KB framework was built with the hypothesis that homologous genes found in collinear regions are most likely to be orthologs. Collinearity mitigates the effects of genome duplication and fractionation and thus most likely pinpoints true orthologs (Tang et al., 2008). Besides bridges between genomes based on orthology, additional information layers were incrementally integrated and connected to gene entities, taking advantage of the modeling flexibility allowed by Neo4j. The integration of such information was planned following homogenization rules for quality purposes. For expression data, we chose to use a single pipeline to process all transcriptomic datasets and avoid prejudice related to discrepancies in bioinformatic analysis protocols including alignment procedure, GC bias treatment. A similar initiative was taken for the gene atlas dedicated to *M. truncatula* (Carrere et al., 2021). We further decided to integrate normalized expression but not differential expression (DE). In fact, since the aim with Ortho_KB is to explore gene expressions across multiple samples and experiments, including expression in the form of DE would restrict analyses to a specific imposed comparison. Yet, the support for differential expression might be provided in the near future. Several actively updated ontologies (PO, PECO) were further selected to best describe the various experimental conditions from which the transcriptomic data were obtained. Since it requires human expertise, the annotation of samples with ontologies remains manual in the current version of the framework.

Regarding QTL, and unlike trancriptomics data, the reprocessing approach in sake of comparability could not be established so far as the analysis requires access to metadata, which are often sparsely provided in the literature. However, as FAIR standards are gaining in popularity, a unified approach might be considered for genetic data analysis in an upcoming version (Wilkinson et al., 2016). To ensure that positions of QTL for similar traits can be compared within and between species, homogeneity in traits denominations is required. This constraint is difficult to meet as a trait can be measured or named differently. For example, the flowering time might be considered by some authors as the time until the first plant has flowered, 50% of the plants have flowered or even 90% of them. Flowering time can also be expressed as the number of days between sowing and flowering or the number of degree-days. Nonetheless, a common vocabulary can be achieved with multi-species ontologies and needs to be developed. Such initiatives exist, such as the BBCH-scale framework to describe the phenological development stages of plants and serialized in RDF (Roussey, 2021), with instances for pea and faba bean but remain under-utilized. In the case of legumes, a higher-level ontology, not restricted to phenological stages, could use existing legume ontologies from the Crop Ontology, including the Lentil Ontology (CO_339) and the Faba bean Ontology (CO_365) (Shrestha et al., 2012). A general, consensus, ontology will however require manual work for the mapping of ontologies and its curation (Oellrich et al., 2015; Laporte et al., 2016; Cooper et al., 2018).

### The graph model of Ortho_KB is intended to be regularly updated to enhance querying possibilities

The current version of Ortho_KB includes QTL and expression data but only allow the comparison of species based on single reference genomes. Lately, efforts on pangenomes and on the description of large diversity panels highlighted the importance of considering a wider set of accessions rather than a single representative one. As a first step toward the integration of structural variation, we intend to upgrade the graph model to allow hosting polymorphism variants in Ortho_KB. Since single nucleotide polymorphism (SNP) matrices constitute a large amount of data, the filtering and modeling will have to be thoroughly tested.

At the functional level, Ortho_KB presently provides solely transcriptomic data evidences. To provide complementary evidences regarding the function of genes of interest and their regulation at the post-transcriptional level, we plan to support the integration of proteomic data with Ortho_KB. This addition is also motivated by the ongoing standardization of proteomics output such as the mzTab format and downstream analyses (Griss et al., 2014; Ewels et al., 2020; Deutsch et al., 2022; Dubbelaar et al., 2022). Indeed, a recently published knowledge graph designed for clinical proteomic data namely the Clinical Knowledge Graph (CKG) accepts community-developed formats including mzTab and SDRF for metadata (Santos et al., 2022). Combining -omics layers can bring further evidence to a hypothesis and also open doors toward the understanding of complex underlying phenomena. Since, Ortho_KB was designed to be modular, one could even consider the inclusion of epigenomics information to gain insights on chromatin rearrangement during stress conditions for example. In this case, the integration of non-genic regions such as promoters and enhancers in the database could be evaluated.

### Ortho_KB should be constantly evaluated to maintain performance and to facilitate its integration in the current databases ecosystem

As more biological data and data types are included, the Ortho_KB framework will have to be regularly fine-tuned to find the optimal graph model, but also in terms of the underlying configuration. In fact, for both the orthology backbone and the additional layers of the graph, single-property indexes have been created on properties that are regularly used as anchors to improve search performance at a small cost in storage space. Further guidance on the configuration of Neo4j has been previously published and will help to ensure high efficiency and scalability of Ortho_KB (Yoon et al., 2017). Several platforms already exist to study comparative genomics (Lyons and Freeling, 2008; Van Bel et al., 2022). The goal of Ortho_KB is different, since it mainly uses orthology and synteny as a way to transfer curated knowledge across species. Therefore, any created instance can be queried freely to answer complex tailored questions in a comprehensive manner.

As OrthoLegKB is primarily populated with published datasets, interoperability with already existing databases is essential. For RNA-seq, the NCBI Sequence Read Archive stores datasets according to defined rigorous standards (NCBI, 2023). QTL data, on the other hand, are typically scattered across multiple databases that store the information in different formats. Unlike the GWAS Catalog available for human (Sollis et al., 2023), no integrative databases store legumes QTL data in a unified format. Therefore, we plan to facilitate the integration of the content from existing legume databases. Other knowledge graph to understand the role of genes are already available. The KnetMiner software was created to analyse genome-scale knowledge graphs, with a recent support for the Cypher graph query language (Hassani-Pak et al., 2021). This platform allows to build gene networks based on semantics and information primarily extracted from the literature, including genetic data, phenotypes associated to SNPs or biological pathways. It was recently applied to wheat, generating networks for the *TT2* gene involved in pre-harvest sprouting (Hassani-Pak et al., 2021). In the specific case of legumes, the AgroLD triplestore is to our knowledge the only phenomics agronomy-centered database aiming at an integrative storage of biological information in the form of a knowledge graph (Venkatesan et al., 2018; Larmande and Todorov, 2021). Since Neo4j can handle RDF import and export, thanks to the neosemantics plugin, data exchange between OrthoLegKB and AgroLD could be considered to take advantage of both technologies. This goal is further supported by the ongoing development of the RDF-star extension which could support properties on edges of the graph (Abuoda et al., 2022). This would bridge the gap between LPG and RDF technologies for improved interoperability (Hartig, 2014). While the SPARQL RDF query language is common to all triplestores, Cypher from Neo4j is only used by the proprietary. However, the open-source GraphQL initiative known as GQL is seen as a potential technology agnostic standardization query language for graph databases (Donkers et al., 2020). We envisage that the legume research community will participate in the data collection and provide feedback on OrthoLegKB for regular improvement.

### Ortho_KB offered an opportunity to develop a valuable tool for translational research in legumes, OrthoLegKB

We decided to select legume species to showcase how the Ortho_KB framework can serve translational research. OrthoLegKB is currently centered on few members mostly diploid cool-season legumes as the identification of orthologs is more straightforward than in polyploid species. Still, having a high-quality assembly is crucial for synteny detection and therefore true orthologs identification. The *FTa* locus in *P. sativum* (*PsFTa*) is in fact incorrectly assembled and annotated in the current version of the Cameor genome. While the *FTa1* gene we identified in *P. sativum* (*Psat3g090720*) is consistent with results from Hecht et al. (2011), the other copy (*Psat3g090680*) was reported as *FTa2* in the same study, both genes displaying similar expression patterns in leaves and apices, but with a weaker expression for *FTa2* (Hecht et al., 2011). In our study, the incomplete annotation of *Psat3g090680* in the version 1 assembly of *P. sativum* cv. Cameor most-likely prevented the creation of orthogroups correctly encompassing the *FT* gene family, and the subsequent inclusion of *PsFTa* in syntenic blocks. The locus displayed increased synteny with the other studied species, this time including *PsFTa1* and *PsFTa2* when considering the more recent *P. sativum* genome assembly from the Zw6 accession (Yang et al., 2022). A new assembly of the Cameor genome is expected soon and should improve the assembly and annotation of this region. Furthermore, supplementing OrthoLegKB with transcriptomic data will provide stronger support when searching for *FT* orthologous genes, by comparing their expression profiles. Fortunately, more legume genomes, have been lately assembled in high-quality using high-throughput chromosome conformation capture sequencing or long-read technologies, namely chickpea (Garg et al., 2022) or common vetch (Xi et al., 2022), which might reveal to be novel sources of data for OrthoLegKB. Thus, the graph will encompass more connected datasets, including information on abiotic and biotic stress response and be useful to a larger part of the legume research community.

### The Ortho_KB framework is for the plant community and beyond

As demonstrated for legumes, the Ortho_KB framework is suitable for translational research within plant families to address common biological questions. Therefore, Ortho_KB could for instance be used in Solanaceae to study late blight attacking potato, tomato but not eggplants nor pepper. Genomes were sequenced for all these diploid species with long-reads technologies (Pham et al., 2020; Wei et al., 2020; Su et al., 2021; Liao et al., 2022). With more caution regarding the identification of orthologs, this resource would also meet the needs of research across plant families, or the needs of polyploids in the Brassicaceae and Poaceae families. Precise study of gene expression bias would be then crucial to identify expressologs (Das et al., 2016). While the scope of Ortho_KB was limited to plants for annotation reasons, its concept could be adapted for the benefit of other communities. For example, the wealth of draft-assembled diploid genomes profiting the Chelicerates community was recently exploited to highlight the conservation of chemosensory genes through comparative genomics (Vizueta et al., 2018). As more qualitative assemblies and associated -omics data are generated across plant and animal groups, we can only anticipate that the need for integrative multi-species databases will increase and Ortho_KB can contribute in this regard.

## Data availability statement

The datasets presented in this study can be found in online repositories. The names of the repositories and accession numbers can be found below in the article/Supplementary material. OrthoLegKB with its user-guide is available for legume translational research at http://ortholegkb.versailles.inrae.fr/browser/. The functional pipeline for synteny is available at: https://forgemia.inra.fr/geapsi/pipeline/specifics_syntenymcscanx. The pipeline to create the translational database is available at: https://forgemia.inra.fr/geapsi/pipeline/specifics_ortho_kb. All scripts used to create the figures presented, including microsynteny from OrthoLegKB data, are available at: https://forgemia.inra.fr/geapsi/ecp-paper/ortholegkb_data.

## Author contributions

BI, JK, and NT contributed to the conception and design of this work. BI was responsible for developing the pipelines, running the use-case, and writing the manuscript. JK and R-GF helped BI to build the graph database and provided bioinformatic support. GA collected information on the datasets to be included in OrthoLegKB and participated in the development of the use-case. JB provided ideas and managed funding acquisition. NT contributed to the scientific management of this work and was involved in drafting and writing the manuscript. All authors participated to manuscript revision, read, and approved the submitted version.
