# Supplementary material for: Development of a knowledge graph framework to ease and empower translational approaches in plant research: a use-case on grain legumes
Source: Front Artif Intell. 2023 Aug 3;6:1191122. doi: 10.3389/frai.2023.1191122 (PMC10435283; doi:10.3389/frai.2023.1191122)
Supplement: Supplementary file 7 [file Data_Sheet_1.docx]

**Supplementary File 1**. Cypher query to traverse syntenic blocks encompassing the *FT* locus and collect included QTL. The QTL "q1" serves as an anchor for the FT locus in *L. culinaris*.

MATCH (q1:QTL)<--(:Gene)-->(sb1:Synteny)-[s1:LOCATED_ON]->(c1:Chromosome)-[:SUBSET_OF]->(g1:Genome) MATCH (c1)<-[s2:LOCATED_ON]-(sb2:Synteny)-->(c2:Chromosome)-[:SUBSET_OF]->(g2:Genome) WHERE q1.qtl_id = "qDTF.6-2_1" AND g1.genome_id in ["mtrun", "psat", "lcul", "vfab"] AND g2.genome_id in ["mtrun", "psat", "lcul", "vfab"] AND ((s2.start >= s1.start AND s2.end <= s1.end) OR (s2.start <= s1.start AND s2.end >= s1.end) OR (s1.start <= s2.start <= s1.end AND s2.end >= s1.end) OR (s2.start <= s1.start <= s2.end AND s1.end >= s2.end)) WITH COLLECT(DISTINCT sb2) as found_blocks UNWIND found_blocks as sb2 MATCH (c1:Chromosome)<-[s1:LOCATED_ON]-(sb2)-[s2:LOCATED_ON]->(c2:Chromosome) WHERE id(c1) > id(c2) WITH sb2 as common_blocks MATCH (c:Chromosome)<-[s:LOCATED_ON]-(q2:QTL)<--(:Gene)-->(common_blocks), (t:Trait)<--(q2) WHERE t.trait_id =~ ".*flower.*" RETURN DISTINCT q2.qtl_id as qtl_id, c.chromosome_id as chr, s.start as start, s.end as end
